# Rural and urban disparities in health-seeking for fever in Myanmar: findings from a probability-based household survey

**DOI:** 10.1186/s12936-016-1442-z

**Published:** 2016-07-25

**Authors:** Tin Aung, Moh Moh Lwin, May Sudhinaraset, Chongyi Wei

**Affiliations:** 1 Population Services International/Myanmar, No. 16, West Shwe Gone Dine 4th Street, Bahan Township, Yangon, Myanmar; 2 Department of Epidemiology and Biostatistics, University of California, San Francisco, Mission Hall 3rd Floor, 550 16th Street, San Francisco, CA 94158 USA

**Keywords:** Health disparity, Myanmar, Malaria, Health-seeking behaviour

## Abstract

**Background:**

The World Health Organization (WHO) recognizes Myanmar as having the highest burden of malaria in the Greater Mekong Sub-region (GMS). Early diagnosis and proper treatment are critical in containing malaria. The objective of this study was to assess determinants of seeking treatment for fever from trained providers across rural and urban areas in Eastern Myanmar.

**Methods:**

A cross-sectional survey was conducted during the high malaria seasons in the eastern part Myanmar between August and September 2014. Multi-staged cluster sampling was used to sample households. A series of questions related to treatment-seeking for fever were asked. Bivariate and multivariate logistic regressions were conducted to identify independent correlates of seeking treatment for fever from trained providers.

**Results:**

The analysis was restricted to 637 participants who reported either themselves or their family members having had fever 2 weeks prior to the interview. In the multivariate analysis, rural residents were less likely to have sought treatment from trained providers (AOR = 0.60, 95 % CI 0.42–0.88; *p* = 0.01) while residents who had fever patients between the ages of 5 and 14 years (AOR = 1.60, 95 % CI 0.90–2.53; *p* = 0.05); and those who knew that sleeping under bed nets can prevent malaria (AOR = 2.08, 95 % CI 1.00–4.30; *p* = 0.05); were borderline more likely to have sought treatment.

**Conclusion:**

This study suggests that rural populations need improved access to trained providers. Additionally, future programmes should focus on increasing knowledge around malaria prevention and treatment.

## Background

Malaria is endemic across ten countries in Southeast Asia, affecting 2.31 billion people [1]. In 2011, 2.7 million cases and over 2000 deaths were reported, where India, Indonesia, Myanmar, and Pakistan accounted for over 85 % of the reported cases and deaths. In the Greater Mekong Sub-region (GMS), the burden of malaria is highest in Myanmar where it remains a significant public health problem. According to the World Malaria Report country profile information of Myanmar, 16 % of Myanmar’s total population lives in high transmission areas (defined as >one case per 1000 population) [1]. Moreover, 62 % of the population in the country lives in malaria-risk areas (21.4 % in high risk, 17.9 % in moderate risk, 22.4 % in low risk areas). According to the Myanmar National Malaria Strategic Plan 2010–2015, the number of malaria cases reported by the National Malaria Control Programme (NMCP) are only confirmed cases where diagnostic services are available [2]. There are a number of villages that still do not have access to malaria diagnosis and treatment, especially in hard-to-reach areas where malaria transmission is probably the highest. In addition, there is a critical lack of data reported from the private sector and the traditional or informal sector. Therefore, the incidence of malaria reported by the NMCP is mostly likely an underestimate.

To prevent malaria, the World Health Organization (WHO) has emphasized that early diagnosis and prompt treatment should occur within 24 h of onset of symptoms to decrease risk of severe complications and onward transmission [3]. Appropriate treatment-seeking behaviour and easy access to health services are important components vital to their success [4]. Specifically, it is recommended that patients should seek medical treatment following the onset of fever, a common symptom of malaria. A few studies that examined treatment-seeking behaviour among people infected with malaria in Bangladesh reported that treatment-seeking behaviour was: associated with: socio-economic status; that children from the poorest families were least likely to seek care from trained providers [5]; proximity to health facilities [6]; accessibility to trained providers [7]; availability of transportation [8]; and, knowledge of malaria, where people who were exposed to mass media or any information sessions were more likely to seek care from trained providers [8, 9].

These barriers to treatment-seeking present special challenges to malaria prevention and control in Myanmar as people at the highest risk and most affected are usually rural and poor. Malaria is endemic in rural areas, where approximately 70 % of Myanmar people live, including living in close proximity to forest and its fringes. Houses located within 1 km of the forest are at particularly high risk. Within Myanmar, the Eastern region has a high disease burden especially among children and this likely constitutes a source of infection for neighbouring regions [10]. It is important to examine health-seeking behaviour for malaria, including its symptoms (i.e., fever is one of the major symptoms of malaria in the region), among rural populations in order to develop better strategies to ensure early testing and treatment and ultimately elimination of malaria in Myanmar. In this paper, determinants of seeking treatment for fever from trained providers across rural and urban areas in Eastern Myanmar were assessed.

## Methods

### Study location

This study was conducted in the Eastern part of Myanmar where malaria is endemic. The study area consisted of 64 townships with an estimated six million population, where Population Services International (PSI) had worked with private sector suppliers and providers to rapidly replace widely available oral artemisinin monotherapy with highly subsidized, quality assured artemisinin-based combination therapy (ACT). At the same time, PSI had invested in massive behavioural change communications (BCC) activities, targeting both consumers and providers, which supplemented the supply chain replacement activities, with the ultimate aim of halting the spread of artemisinin-resistant *Plasmodium falciparum* in the region. As a part of a larger evaluation study, this survey was conducted among the 64 townships where PSI carried out the above intervention activities.

In Myanmar there are 14 states/regions, each comprised of 20–25 townships. Township level is the most common administrative unit, and there are 330 townships. A typical township consists of a small urban area/town and vast rural area. All the township level administrative bodies and structures are situated in urban areas. Urban areas consist of three to ten wards, while rural areas consist of 15–30 village tracts. Usually the urban area of a township has one urban health centre, one township hospital, a number of general-practitioner clinics, pharmacies, and general retail shops. The rural area has one rural health center per four to five village tracts, a sub-centre (health) per village tract, many community health workers, and general retail shops.

### Study design and recruitment

**Fig. 1:**
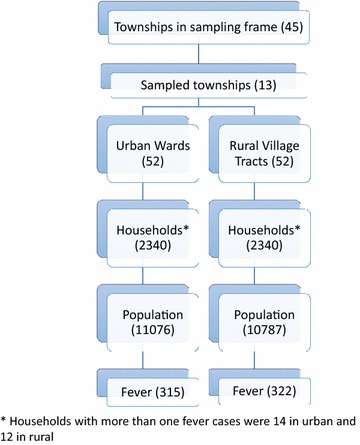
Flow diagram showing sampling procedure

A cross-sectional survey was conducted during the malaria high seasons in eastern Myanmar between August and September 2014. Using multi-staged cluster sampling, 13 townships among the 64 were selected, using probability population to size (PPS). In each selected township, four wards and four village tracts were selected with PPS. In Myanmar, each township is composed of an urban area which is sub-divided into wards, and a rural area which is sub-divided into wider village tracts where an estimated 70 % of population lives. The survey team mapped households in each selected township and 45 households in each cluster were selected using systematic random sampling. Among the 4680 households selected, household members or their family members who had had fever in the previous 2 weeks were interviewed. Specifically, the respondents were asked if they suspected the fever was malaria related. As malaria is well known in the study area, people generally first think of malaria whenever they have fever, especially when it is accompanied with chills and rigours. Of 640 household members who had fever, three declined interview (decline rate = 0.46 %) (Fig. 1). The respondents were the caregivers if the fever cases were under 15 years old. Trained interviewers explained the purpose of the study to each respondent and obtained verbal consent from a representative of the household, who was either the head or spouse of the head to be interviewed. When those people were not available, one of the adult members of the household was selected to be interviewed. Face-to-face interviews were conducted by trained interviewers with structured questionnaires and were conducted either in Myanmar or a local language.

### Measures

Demographic information, including age and gender of fever cases, education, and occupation of main household earner, and the total number of household members, was collected. Ownership of household assets was asked to calculate the wealth quintile. Questions of health-seeking behaviour for fever suspected of malaria included if and where treatment was sought, timing of seeking treatment (within vs over 24 h), primary reasons of choosing providers or not seeking treatment, waiting time at provider, estimated amount of money spent for treatment, perception of provider’s dealing, satisfaction with provider, and knowledge of malaria diagnosis and treatment. Respondents were also asked what types of providers they sought for themselves or their family member when they had fever. In Myanmar, the following types of facilities/providers were considered as trained: (1) public health facilities, including public hospitals, township health centres; (2) private health facilities, including health providers from worksites, general practitioners, and Sun Quality health clinics (SQH), a social, franchised network of 1400 private general practitioners’ clinics in Myanmar); and (3) community health workers. Retail outlets such as pharmacies that are licensed to sell drugs, drug stores which are not licensed, grocery or village shops where people can buy drugs, and itinerate drug vendors, informal providers and ‘quacks’ are considered untrained.

### Data analysis

CSPro software was used to enter data and the datasets were later converted to STATA version 13.0 for analysis. Chi square tests were conducted to compare differences in socio-demographic characteristics and treatment-seeking behaviour between urban and rural households. To identify independent correlates of seeking treatment for fever from trained providers, bivariate analysis was first conducted where correlates with *p* values less than 0.2 were entered into a multivariate logistic regression model. The independent variables included in the final model were urban/rural residency, age of fever cases, education of the main household income earner, occupation of the main earner and malaria knowledge of fever cases or caregivers if the fever case happened to be under 15 years of age. The multivariate model did not include wealth quintile since it was collinear with urban/rural residency.

### Ethical consideration

All respondents were informed of the purpose of the study. The respondents were fully aware that they had the right not to participate or to withdraw from the study at any time. Confidentiality was maintained at all steps of data collection. Participation was entirely voluntary and verbal consent was obtained from all respondents. The project obtained ethical approval from PSI Ethical Review Board.

## Results

Between August and September 2014, 4680 households were sampled as part of a larger study, with a total of 21,861 household members. Among those, 637 (2.9 %) respondents reported that either they or one of their family members had had fever two weeks prior to the interview. Compared to urban residents, rural residents who had had fever were more likely to be between the ages of 15 and 45 (30.8 vs 23.2 %, *p* = 0.033) (Table 1). Fever patients’ household characteristics differed significantly as well. Compared to urban households, rural fever patients were more likely to have a main income earner with no formal schooling (25.2 vs 9.5 %, *p* < 0.001), a main income earner who was a farmer or fisherman (51.6 vs 22.5 %, *p* < 0.001), and had lower household socio-economic status (SES) (e.g., 30.4 vs 13.3 % at Level 1, *p* < 0.001). In terms of health-seeking behaviour, rural residents were less likely to have sought treatment outside of their homes (69.3 vs 82.2 %, *p* < 0.001) or have sought treatment within 24 h of fever onset (50.9 *vs* 59.4 %, *p* = 0.032). Among those who were treated at home, rural residents were more likely to use home remedies than urban residents (9.0 vs 2.9 %, *p* = 0.001). Knowledge of malaria did not appear to differ significantly between urban and rural residents, except that rural residents were less likely to know that malaria is transmitted through mosquito bites (64.3 vs 72.1 %, *p* = 0.035).

**Table 1:** Demographic characteristics, health-seeking behaviour and health knowledge among individuals with fever 2 weeks prior to interview, Myanmar (N = 637)

Characteristics	Urban (N = 315)	Rural (N = 322)	Total (N = 637)	*p* value
Age of fever cases (years)				0.033
Under 5	65 (20.6)	79 (24.5)	144 (22.6)	
5–14	100 (31.8)	84 (26.1)	184 (28.9)	
15–45	73 (23.2)	99 (30.8)	172 (27.0)	
46 and above	77 (24.4)	60 (18.6)	137 (21.5)	
Gender of fever cases				0.688
Male	138 (43.8)	136 (42.2)	274 (43.0)	
Female	177 (56.2)	186 (57.8)	363 (57.0)	
Education of main income earner				0.000
No schooling	30 (9.5)	81 (25.2)	111 (17.4)	
Primary grade	104 (33.0)	152 (47.2)	256 (40.2)	
Middle grade	95 (30.2)	60 (18.6)	155 (24.3)	
High grade and above	86 (27.3)	29 (9.0)	115 (18.05)	
Occupation of main income earner				0.000
Professionals/skilled workers	38 (12.1)	9 (2.8)	47 (7.4)	
Farmers/fishermen	71 (22.5)	166 (51.6)	237 (37.21)	
Self-employed/shop owners	89 (28.3)	22 (6.8)	111 (17.43)	
Unskilled labourers	117 (37.1)	125 (38.8)	242 (38.1)	
Household SES quintile				0.000
Level 1	42 (13.3)	98 (30.4)	140 (22.0)	
Level 2	43 (13.7)	77 (23.9)	120 (18.8)	
Level 3	63 (20.0)	79 (24.5)	142 (22.3)	
Level 4	72 (22.9)	42 (13.0)	114 (17.9)	
Level 5	95 (30.2)	26 (8.1)	121 (19.0)	
Had additional symptoms (multiple responses)	155 (24.3)	130 (20.4)	285 (44.7)	0.025
Cough	68 (21.6)	64 (19.9)	132 (20.72)	0.594
Aches and pains	52 (16.5)	41 (12.7)	93 (14.6)	0.177
Sweating	11 (3.5)	2 (0.6)	13 (2.04)	0.010
Chills	11 (3.5)	17 (5.3)	28 (4.4)	0.271
Vomiting	11 (3.5)	8 (2.5)	19 (3.0)	0.455
Less active	10 (3.2)	8 (2.5)	18 (2.8)	0.599
Loss of appetite	9 (2.9)	8 (2.5)	17 (2.7)	0.770
Treated at home	61 (19.4)	98 (30.4)	159 (25.0)	0.001
Home remedies	9 (2.9)	29 (9.0)	38 (6.0)	0.001
Traditional medicine	18 (5.7)	21 (6.5)	39 (6.1)	0.671
Modern medicine	34 (10.8)	48 (14.9)	82 (12.9)	0.121
Sought treatment outside	259 (82.2)	223 (69.3)	482 (75.7)	0.000
Early seeking treatment				0.032
Sought treatment within 24 h of fever onset	187 (59.4)	164 (50.9)	351 (55.1)	
Sought treatment >24 h after fever onset	128 (40.6)	158 (49.1)	286 (44.9)	
Malaria knowledge				
Know that mosquito bite can cause malaria	227 (72.1)	207 (64.3)	434 (68.1)	0.035
Think malaria is severe in community	55 (17.5)	66 (20.5)	121 (19.0)	0.329
Know sleeping under bed net can prevent malaria	16 (5.1)	25 (7.8)	41 (6.4)	0.167
Know of at least one anti-malarial drug	60 (19.1)	48 (14.9)	108 (17.0)	0.164
Heard of rapid diagnostic testing	49 (15.6)	58 (18.0)	107 (16.8)	0.407

Among those who sought treatment outside of their homes, urban people were more likely to seek treatment at trained providers than rural residents (65.1 vs 51.6 %, *p* < 0.001) (Table 2). In terms of primary reasons for choosing a provider, the most commonly cited reason for both urban and rural respondents was that providers were close by or easy to get to. However, urban people were more likely than rural residents to choose providers based on reputable quality service (23.6 vs 13.9 %, *p* < 0.001). Patients from urban households were more likely to perceive that the price spent at providers was expensive or very expensive compared to rural people (45.9 vs 30.5 %, *p* = 0.001). Over 90 % of both urban and rural respondents reported that they felt providers treated them with respect and were friendly. Among those who did not seek treatment outside of their homes, the most commonly cited reason was that their fever went away quickly (71.5 %).

**Table 2:** Reasons for choosing providers, services received and satisfactory level to among individuals having fever two weeks prior to interview in Myanmar (N = 637)

	Urban (N = 315)	Rural (N = 322)	Total (N = 637)	*p* value
	Urban (n = 259)	Rural (n = 223)	Total (n = 482)	
*Sought treatment from providers*				
Provider type				0.000
Trained providers	205 (65.1)	166 (51.6)	371 (58.2)	
Untrained providers	54 (34.9)	57 (48.4)	111 (41.8)	
Primary reason for choosing provider				0.218
Close by/easy to reach	158 (61.0)	158 (70.9)	316 (65.6)	0.783
Reputable quality service	61 (23.6)	31 (13.9)	92 (19.1)	0.000
Availability of inexpensive treatment	15 (5.8)	11 (4.9)	26 (5.4)	0.391
Availability of modern medicine	7 (2.7)	6 (2.7)	13 (2.7)	0.749
Speed of treatment	9 (3.5)	6 (2.7)	15 (3.1)	0.408
Can handle severe cases	6 (2.3)	7 (3.1)	13 (2.7)	0.810
Employer arranged	0 (0.0)	1 (0.5)	1 (0.2)	0.322
Other	3 (1.2)	3 (1.4)	6 (1.2)	0.978
Waiting time				0.756
Long and very long	38 (14.7)	26 (11.7)	64 (13.3)	
No or short waiting time	221 (85.3)	197 (88.3)	418 (86.7)	
Perception on price				0.001
Very inexpensive	68 (26.3)	91 (40.8)	159 (32.9)	
Somewhat inexpensive	72 (27.8)	64 (28.7)	136 (28.2)	
Expensive to very expensive	119 (45.9)	68 (30.5)	187 (38.9)	
Service received				
Received test for malaria	19 (7.3)	16 (7.2)	35 (7.3)	0.648
Received modern medicine^a^	253 (97.7)	217 (97.3)	470 (97.5)	0.793
Received antibiotic	15 (4.7)	9 (2.8)	24 (3.8)	0.192
Received injection	63 (20)	46 (14.3)	109 (17.1)	0.056
Provider dealing				0.302
Friendly and respectful	251 (96.9)	215 (96.4)	466 (96.7)	
Unfriendly, rude or not sure	8 (3.1)	8 (3.6)	16 (3.3)	
Overall satisfaction				0.255
Very satisfied	192 (74.1)	177 (79.4)	369 (76.6)	
Somewhat satisfied	61 (23.6)	43 (19.3)	104 (21.6)	
Dissatisfied	5 (1.9)	1 (0.5)	6 (1.2)	
Don’t know	1 (0.4)	2 (0.9)	3 (0.6)	

	Urban (n = 56)	Rural (n = 99)	Total (n = 155)	
*Reasons for not seeking treatment*				
Thought that fever is not serious	15 (26.8)	25 (25.3)	40 (25.8)	0.834
Because fever went away	38 (67.9)	72 (72.7)	110 (71.0)	0.521
No money for treatment	0 (0.0)	4 (4.04)	4 (2.6)	0.128
No transportation	1 (1.8)	0 (0.0)	1 (0.7)	0.182
The places were too far away	1 (1.8)	0 (0.0)	1 (0.7)	0.182
Wait and see	2 (3.6)	8 (8.1)	10 (6.5)	0.272

^a^ Modern medicine includes tablets, capsule syrups, injections, and antibiotics

In the multivariate analysis (Table 3), rural residents were less likely to have sought treatment from trained providers (AOR = 0.60, 95 % CI 0.42–0.88; *p* = 0.01) while residents who had fever patients between the ages of five and 14 years (AOR = 1.60, 95 % CI 0.90–2.53; *p* = 0.05) and those who knew that sleeping under bed nets can prevent malaria (AOR = 2.08, 95 % CI 1.00–4.30; *p* = 0.05) were borderline more likely to have sought treatment from trained providers.

**Table 3:** Multivariate correlates among people who sought treatment for fever from trained providers in Myanmar (N = 637)

	Sought treatment from trained providers		
	Adjusted odds ratio	95 % CI	*p* value
Residence			
Urban	1		
Rural	0.60	0.42–0.88	0.01
Age (years)			
Under 5	1		
5–14	1.60	0.90–2.53	0.05
15–45	0.87	0.55–1.38	0.55
46 and above	0.78	0.48–1.27	0.32
Education			
Primary grade	1		
No schooling	1.13	0.70–1.82	0.62
Middle grade	1.09	0.71–1.69	0.69
High grade and above	1.02	0.61–1.73	0.93
Occupation			
Professionals/skilled workers	1		
Farmers/fishermen	0.86	0.41–1.77	0.68
Self-employed/shops	1.05	0.50–2.20	0.89
Unskilled labourers	0.70	0.35–1.42	0.33
Others			
Had additional symptoms	1.32	0.94–1.85	0.10
Had known that mosquito bite can cause malaria	1.02	0.71–1.46	0.93
Think that malaria is severe disease	0.96	0.63–1.46	0.84
Know that sleeping under bed net can prevent malaria	2.08	1.00–4.30	0.05
Heard of rapid diagnostic testing	1.22	0.78–1.93	0.39

## Discussion

This was one of the first studies to document health-seeking for fever among rural and urban populations in Myanmar. This study provided evidence that there was a significant disparity in seeking care from trained providers between urban and rural populations in Myanmar. This could be due to the fact that a majority of health facilities are located in urban areas [11]. Furthermore, the ratio of health facilities to population size is significantly different between rural and urban areas. According to the Myanmar Health System Strengthening Review Report, there was evidence that expansion of rural health centres (RHCs) increased to 4 % in 2012 compared to 2011; however, there was a downward trend in 2013 and 2014 [11]. On the other hand, hospitals in urban areas increased from 897 in 2010, 944 in 2012, to 1065 in 2014. In addition to lack of health facilities in rural areas, a significant proportion of respondents who sought treatment also thought that it was either expensive or very expensive. Among those who did not seek treatment outside of their homes, about a quarter thought that fever was not serious. Combined, these factors were significant barriers to accessing trained providers for rural populations and present challenges for early detection and treatment for malaria.

It was also found that those who had greater knowledge of malaria transmission and prevention were over two times more likely to seek care from trained providers than those who did not have sufficient knowledge. Nyunt et al. also found that correct knowledge on causes of malaria was associated with increased use of health care centres [9]. Future efforts should focus on improving knowledge for malaria treatment and prevention, particularly in rural areas.

Contrary to intuition, caregivers who had older children with fever (5–14 years old) were more likely to see a trained provider compared to those who had younger children with fever (under 5 years old). A potential explanation for this finding could be that younger children tended to get sick more often than older children and parents tended to wait and try with some home remedies before seeking treatment outside.

There are several limitations to this study. First, some providers might have been misclassified in the analysis. The providers were categorized into trained vs untrained as reported by individuals suffering from fever or in the case of children, the caregiver of a sick child. In actuality, respondents may not know whether the provider was formally trained or not. However, the way the provider types were categorized is well established in Myanmar. Second, this study was conducted in eastern Myanmar and may not be generalizable to populations in other parts of country. Finally, the questionnaire asked about treatment-seeking behaviour for fever suspected of being malaria and was not able to confirm that respondents were referring to malaria fever only.

## Conclusion

Despite the limitations, this study demonstrated that there were significant disparities in health seeking for fever between rural and urban populations in Myanmar. The findings have important implications for malaria prevention and improving the general health among rural populations in Myanmar. First, more health centres should be established and staffed with trained health workers in rural areas. These centres should be easily accessible to the most affected groups and charge lower fees for treatment. Health workers should be trained to be able to detect malaria fever early and provide proper treatment. Second, BCC campaigns, including mass media to disseminate behavioural change messages and peer-to-peer, small-section health talks, should be ongoing and tailored to target sub-groups most at risk of malaria yet who lack knowledge of malaria prevention. Finally, a holistic approach to improving the health of rural populations should be adopted where infectious diseases, such as malaria, should not be the only focus.

## Abbreviations

ACT: artemisinin-based combination therapy
BCC: behavioural change communications
GMS: Greater Mekong Sub-region
NMCP: National Malaria Control Programme
PPS: probability population to size
PSI: Population Services International
SES: socio-economic status
RHC: rural health centre
SQH: Sun Quality health clinics
WHO: World Health Organization
